# Analysis of the top coal stability of the large section open-off cut under the gob in thick seams slicing mining

**DOI:** 10.1038/s41598-022-21066-x

**Published:** 2022-10-05

**Authors:** Jing Chai, Yongliang Liu, Shigang Gao, Jianfeng Yang, Dingding Zhang, Wengang Du, Chenyang Ma, Zhicheng Han

**Affiliations:** 1 College of Energy Engineering, Xi’an University of Science and Technology, Xi’an, 710054 China; 2 Shendong Coal Group Corporation Limited, China Energy Group, Shenmu, 719315 China

**Keywords:** Engineering, Civil engineering

## Abstract

The reserved thickness of top coal has an important influence on the stability of a large section open-off cut under gob in the thick seams slicing mining. The destabilization extremum conditions of the open-off cut top coal were derived from by elastic–plastic theory, and the optical fibre sensing technology was utilized to monitor the top coal deformation law with different thicknesses (3, 3.5, and 4 m) in the physical similar simulation experiment in the paper. The results show that the top coal thickness is greater than 3.4 m without tension cracks. In the vertical direction, the top coal of the large open-off cut is divided into mining and excavation disturbance zones under the influence of the upper slice coal mining and the excavation disturbance. In the direction of the span of the top coal can be divided into the roof fall risk zone and the warning zone. The deformation changes from exponential to linear to logarithmic in the roof fall risk zone, and it changes from linear to logarithmic in the roof fall warning zone as the number of excavations increases. The sinking amount in the two zones is smaller as the thickness of the top coal becomes larger. It is comprehensively determined that the thickness of the top coal of open-off cut is set as 3.5 m, the stability is moderate, and the field application shows that the integrity of the top coal is good after support, and the maximum off-layer value is 6 mm, which can satisfy the production requirements.

## Introduction

The maximum minable thickness of the coal seam is up to 12.5 m in the Yushenfu mining area in Northern Shaanxi, China. Due to the limitation of the technical level, layered mining technology is adopted for mining in the early stage of resource development^1–3^. Influence of nearby small kiln cross-border mining and geological structure and other factors, the open-off cut has to be laid below the mining gob when the lower slice coal resources mining. With the increase in mine production and the enlargement of fully mechanized mining equipment in recent years, the section of the open-off cut has continuously enlarged, and it is more difficult to control the stability of the surrounding rock^4–6^.

To analyze the stability of the open-off cut roof, scholars simplified the roof as a beam through the method of theoretical analysis to investigate the stability^7,8^. The mechanical structure model of the continuous beam under a uniformly distributed load was established by Jiang et al. to obtain the stress distribution law in the cut roof rock layer under roadway mining^9^. The maximum deflection and rotation angle of mining roadway under three different combined structures of roadway broken roof rock mass beam, fixed support beam, and the composite beam was deduced by Yao et al. and discussed the relationship between roadway width, interlayer thickness, mining height of upper coal seam and roof deformation^10^. The mechanical model of fracture instability of long-span open-off cut was proposed by Jia et al. and analyzed the instability and collapse form of the roof^11^.

As for the deformation law and support technology of the open-off cut surrounding rock, He et al. conducted a systematic study on the relationship between the width of the open-off cut and stress and deformation. The result shows that the larger the width, the smaller the shallow partial stress in the roof of the cut, the larger the deep partial stress, and the value of partial stress gradually decreases and shifts to the deep. The peak of the main stress difference in the roof is constant and then decreases, it is first decreased and then constant on the floor. there is a gradually increasing trend in the two sides of the open-off cut hole^12^. The fracture field of the surrounding rock of the open-off cut is divided into fracture penetration zone, fracture development zone, and microfracture zone. In addition, with the increase in the width of the open-off cut , the evolution process from the microfracture zone to the fracture development zone and the fracture development zone to the fracture penetration zone of the roof is promoted^13^. Furthermore, the top coal plastic zone is rectangular when the thickness of the top coal is small 6 m, it is arch-shaped when the thickness is larger than 6 m. With the increase of the width of the open-off cut, the roof pulling damaged area increases in an approximately linear relationship, and the deformation dividing point of a fast and slow decline in the top coal gradually rise^14^.

Zhao et al. found that the range of the plastic zone of the surrounding rock is expanding with the increased width of the roadway, and the displacement on the two sides of the roadway changes slowly, but roof falling capacity increases significantly, and the location of maximum deformation appears in the middle position of the roof^15^. Wang et al. studied the deformation characteristics of the surrounding rock of the large section open-off cut with a composite roof and found that the top and bottom locations of the open-off cut and the roof are easy to damage, and the damage during the expansion of the open-off cut significantly exceeds the period of the excavating^16,17^.

In the aspect of large section open-off cut support technology, Sun et al. studied the support technology of open-off cut under compound roof conditions of large buried depth three soft coal seams, and proposed the joint support technology of high-strength anchor rod anchor network add long and short anchor cable joist to reinforce the surrounding rock, which achieved a good effect of surrounding rock control^18^. He et al. researched the reasonable configuration of anchor cables for large cross-sectional open-off cut support and proposed the "cross-stepping" joint control technology, which was successfully applied in the field. Jia et al. proposed a roof zoning control technology with the anchor rod support, and clarified the control principle of different roof zoning for the problems of poor self-stabilization, violent deformation and difficult support of deep large-span open-off cut roof^19^. In order to solve the problem of roof support for soft and thick coal seams in extra-thick sections, Yan Hong et al. proposed a control system of twice forming the roadway with evenly divided sections and "multiple support structures", and calculated the safety zoning and safety evaluation factors of the roof of extra-thick coal seams^20^. A numerical model was proposed to investigate the stability of the Angouran underground mine batching room, design appropriate support systems, and to forecast potentially dangerous rock pressure manifestations in the mine roadways^21–24^.

The optical fiber sensor has been widely used in mine safety monitoring. The roof internal displacement sensor with a range of 50 mm and a displacement resolution of 0.06 mm was made to monitor the roof stability by connecting the FBG with a spring mechanism^25–27^. A temperature-compensated strain sensor composed of 12 fiber Bragg grating sensors was proposed by GAGE et al. which was implanted in the roadway roof to realize dynamic monitoring of roof deformation^28^. The temperature compensated roadway deformation online monitoring system based on the fiber Bragg grating sensor was established, and the system realized the real-time dynamic and continuous monitoring of the precursor information of mining roadway deformation damage, which provided a reference for the online monitoring of roadway stability^29^. In addition, the distribution optical fiber sensing technology was utilized for roadway deformation monitoring, realizing the deformation monitoring of the roadway roof and two sides by deploying distributed optical fibers between roadway anchors and cables and in the lining^30,31^. The achievements are to confirm the applicability of optical fiber sensing technology in the mining field.

Overall, there are more findings on the deformation of large section open-off cuts. But the influence of the top coal thickness on the stability of the roof for large section open-off cut under slicing mining conditions is few reports. The roof stability of large section roadway under gob has become one of the difficult problems in many minefields of China. To ensure safety and as much as possible to recover coal resources, determining reasonable top coal thickness has become the bottleneck restricting the safe and efficient production of mines under analogous conditions. Thus, it is favorable to enhance the recovery rate of coal resources and the safe and efficient production of mines by researching the reasonable thickness of the top coal in the large section under the gob.

To determine the reasonable top coal thickness of large section open-off cut under gob and the deformation law of different thickness of top coal in the process of excavation, the methods of theoretical calculation and physical similarity simulation experiment were utilized to study the reasonable thickness of top coal in this paper. The optical fiber sensing technology was used to monitor the deformation law of top coal with different top coal thicknesses. The results would provide the scientific basis for the retention of top surface coal under analog conditions.

## Calculation of the reasonable top coal thickness

### Geological and mining conditions overview

**Figure 1:**
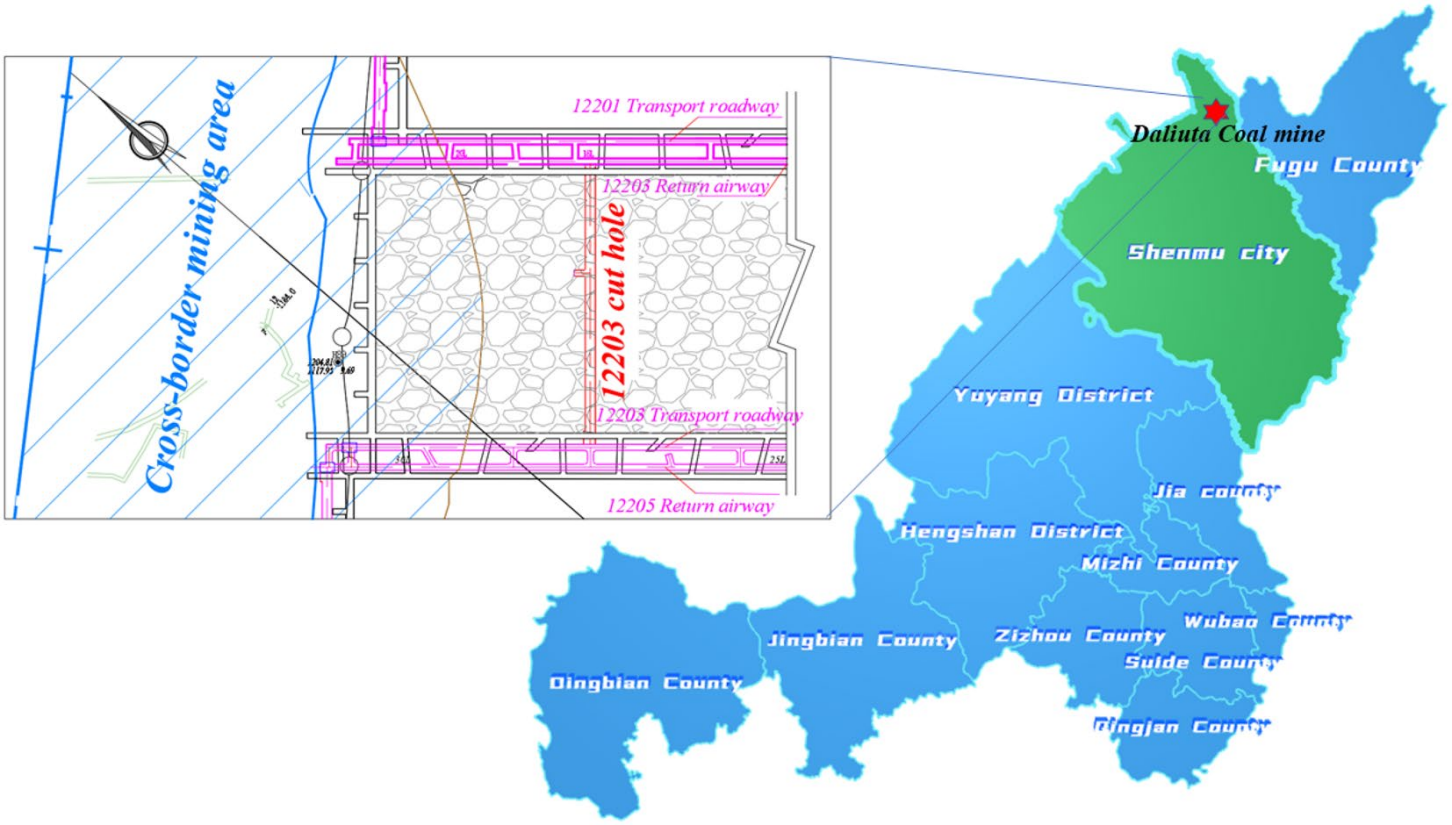
The location of working face 12,203 of Huojitu minefield, Shaanxi, China.

Daliuta coal mine is a super large modern mine located in Daliuta Town, Yulin City, Shaanxi Province, China. It is composed of Daliuta and Huojitu minefield with an annual output of 33Mt. The average coal thickness and the buried depth of 1–2 coal of the Huojitu minefield are 10 m and 76 m. Due to the large thickness of the coal seam and the limitation of mining equipment in the early years, the layered mining method was applied in the 1–2 coal. The design mining height of the upper layer is 4.5 ~ 5 m. The remaining 5 m thick coal seam is retained under the upper layered gob, and the coal resources remain exceed 30 million tons in the area. The lower 12,203 open-off cut is arranged at the lower part of the gob 146 m southeast of the upper 12,203 open-off cut affected cross-boundary mining of small coal kiln of 2–2 coal. It is 9 m wide, 3.9 m high, and 251.4 m long, (shown in Fig. 1). The total excavation quantity is up to 302.4 m. The top coal thickness is increased by cutting the floor in the excavation process, and the support is strengthened to ensure the stability of the top coal to prevent the open-off cut from connecting with the upper gob. Therefore, determining the reasonable reserved thickness of top coal is the core problem to ensure large section cutting under similar conditions.

### The internal force distribution of top coal

**Figure 2:**
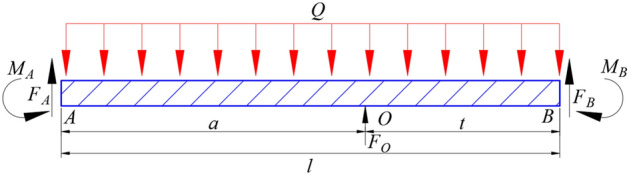
Loading form of top coal.

Based on the top coal shape and supporting stress characteristics of the lower12203 open-off cut , the top coal of the open-off cut can be simplified as two span fixed support beam, as shown in Fig. 2.

In Fig. 2, the resultant force of fulcrum reaction and single hydraulic strut is *F*_*A*_ and *F*_*B*_. The net load on top coal is *Q*, *MPa*. *l* presents the width of the roadway, m; *F*_*B*_ is the support reaction force of single hydraulic prop, *KN*. *M*_*A*_, *M*_*B*_ is the bending moment in the roadway side, *KN·m*. *a* is the distance between left side of roadway and single hydraulic strut, *m*. *t* is the distance between right side of roadway and single hydraulic prop, *m*.

The unit step function^32^ is introduced to calculate the distributed internal force along the width direction of open-off cut.

1 $$ \mu \left( {{\text{x}} - \xi } \right) = \left\{ {\begin{array}{*{20}l} 0 \hfill & {\left( {{\text{x < }}\xi } \right)} \hfill \\ 1 \hfill & {\left( {{\text{x}} \ge \xi } \right)} \hfill \\ \end{array} } \right. $$

**Figure 3:**
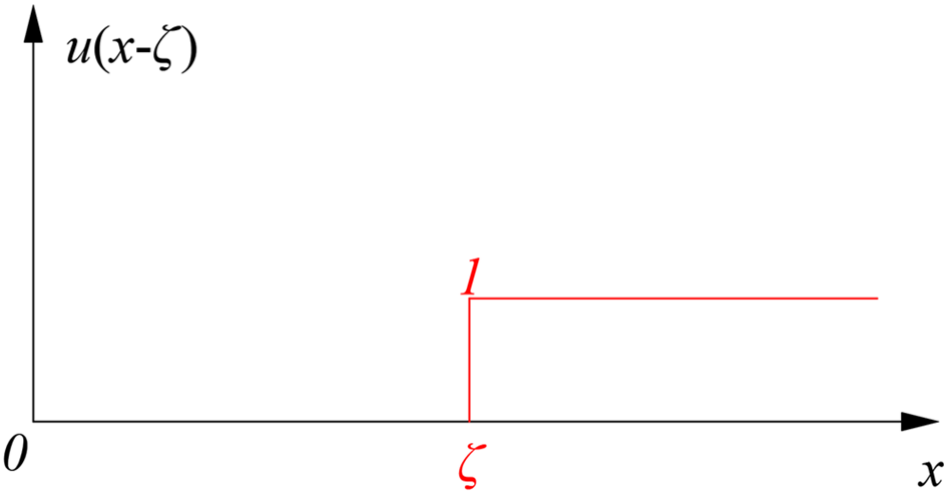
The unit step function.

The unit step function is represented by the step polyline in Fig. 3:

Based on the unit step function, the bending moment, rotation angle and deflection equations of the top coal continuously distributed in the width direction of the open-off cut are to be obtained.

2 $$ EI\frac{{d^{2} \omega }}{{dx^{2} }} = M_{A} + F_{A} x + \mu \left( {x - a} \right)F_{o} t - \frac{1}{2}Qx^{2} $$

3 $$ EI\frac{d\omega }{{dx}} = M_{A} x + \frac{1}{2}F_{A} x^{2} + \mu \left( {x - a} \right)\frac{1}{2}F_{o} t^{2} - \frac{1}{6}Qx^{3} + C $$

4 $$ EI\omega = \frac{1}{2}M_{A} x^{2} + \frac{1}{6}F_{A} x^{3} + \mu \left( {x - a} \right)\frac{1}{6}F_{o} t^{3} - \frac{1}{24}Qx^{4} + Cx + D $$

According to the boundary conditions: when $x = 0$ or ${{x = l}}$: $\omega^{\prime} = 0$, $\omega = 0$, substituting the results into Eqs. (3) and (4), and the solution is: ${\text{C}} = 0$, ${\text{D}} = 0$; At the same time, *M*_*A*_ and *F*_*A*_ are to be got, and the results are substituted into Eq. (2), and the bending moment distribution equation along the width direction of top coal is obtained:

5 $$  M\left( x \right) = \left\{ {\left| \begin{gathered}   \frac{1}{l}F_{o} t^{2}  - \frac{1}{{l^{2} }}F_{o} t^{3}  - \frac{1}{{12}}Ql^{2}  \hfill \\    + \left( {\frac{2}{{l^{3} }}F_{o} t^{3}  - \frac{3}{{l^{2} }}F_{o} t^{2}  + \frac{1}{2}Ql} \right) x  \hfill \\    - \frac{1}{2}Qx^{2}  + \mu \left( {x - a} \right)F_{o} t   \hfill \\  \end{gathered}  \right|} \right.   $$

Based on ${\text{F}}_{{\text{S}}} { = }\frac{{{\text{dM}}\left( {\text{x}} \right)}}{{{\text{dx}}}}$, The shear equation of is obtained

6 $$  F_{s} \left( x \right) = \left| \begin{gathered}   \frac{2}{{l^{3} }}F_{o} t^{3}  - \frac{3}{{l^{2} }}F_{o} t^{2}  + \frac{1}{2}Ql - Qx \hfill \\    + \mu \left( {x - a} \right)F_{o}  \hfill \\  \end{gathered}  \right| $$

### The extreme conditions of top coal instability

**Figure 4:**
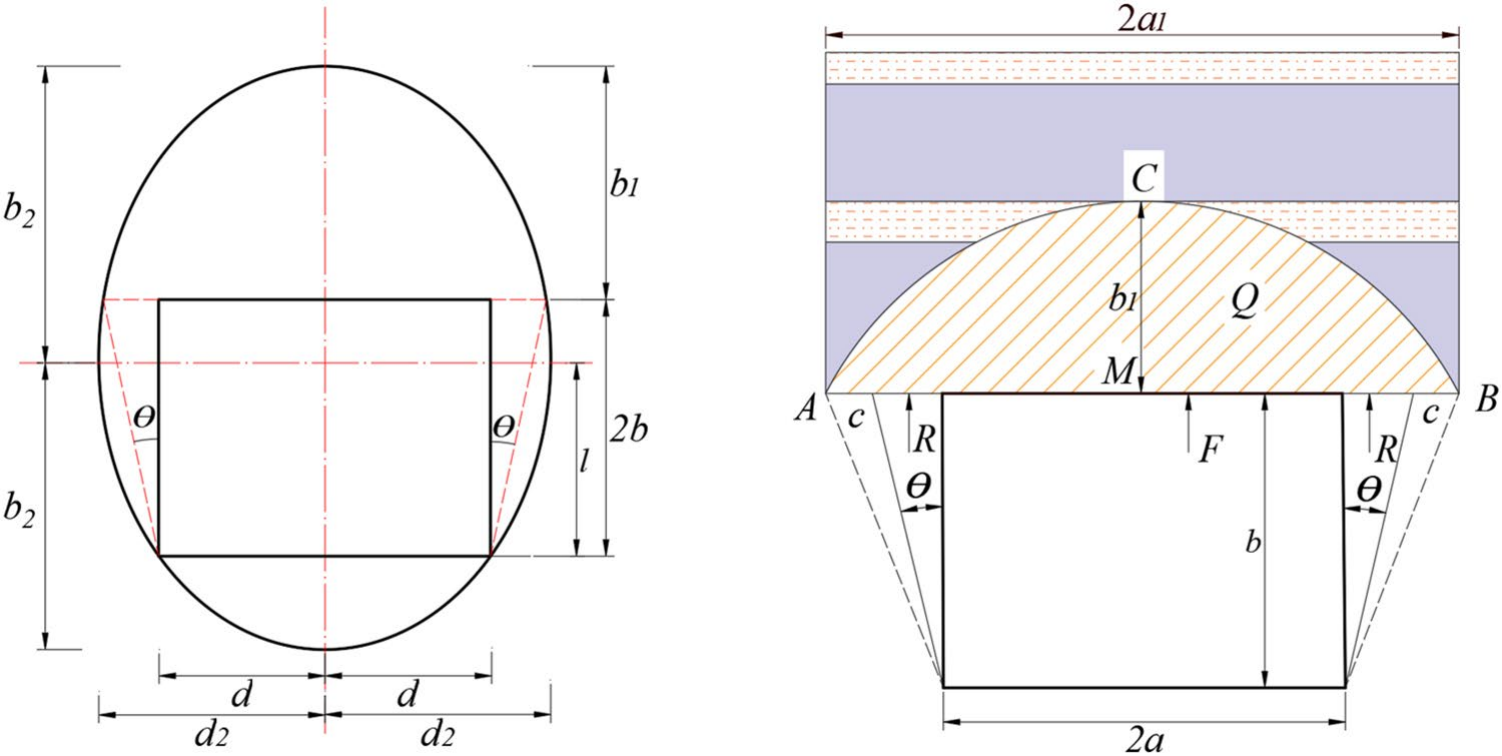
Relative position of limit equilibrium arch and roadway.

Based on the principle of natural equilibrium arch and limit equilibrium^33–35^, the equilibrium state of the original rock stress of the road is destroyed during roadway excavation, and the stress is redistributed. The limit equilibrium is reached when the collapse angle $\theta = \frac{\pi }{4} - \frac{\varphi }{2}$. It is not considering the support influence; a limit pressure cycle is to be formed along with a certain range around the roadway. The relative position relationship between the loop line and the roadway is shown in Fig. 4.

The self weight stress of rock in arch can be obtained in the formular (7):

7 $$  Q_{k}  = \gamma \left( {l + \frac{{d_{2} }}{{\sqrt \lambda  }} - 2b} \right)  $$

The collapse arch height is:

8 $$  b_{1}  = l + \frac{{d_{2} }}{{\sqrt \lambda  }} - 2b  $$

In the above formular: ${{l}} = \frac{{{\text{d}}\tan \theta + {\text{b}}\left( {\lambda + \tan^{2} \theta } \right)}}{\lambda }$, the length of short half axis of pressure arch is *d*_*2*_, $d_{2} = \sqrt {d^{2} + {\text{l}}^{2} }$, the collapse angle is $\theta = \frac{\pi }{4} - \frac{\varphi }{2}$, the average unit weight of rock in the collapse zone is $\gamma = 23$ KN/m^3^, *φ* is the coal friction angle, taken as 28°, $\lambda$ is the side pressure coefficient, taken as 0.8, *d* is half of the width of the open-off cut , taking 4.5 m; *2b* is the cutting height, taken as 3.9 m.

The parameters mentioned are substituted into Eqs. (7), (8). When the top coal is not considered, the results are calculated as follows: the caving rock load *Q*_*k*_ in the gob and the arch height *b*_*1*_ are 0.25 MPa and 10.9 m.

Clearly, the arch height is much greater than the traditional anchor cable support length. Therefore, reserving appropriate top coal thickness, giving full play to the active support role of bolt (cable) and improving the bearing capacity of top coal are the key to ensure the roof stability of the lower open-off cut.

The bending moment and shear force distributed along the width of top coal is to be got by taking *Q* = *Q*_k_ = 0.25 MPa into Eqs. (5) and (6).

**Figure 5:**
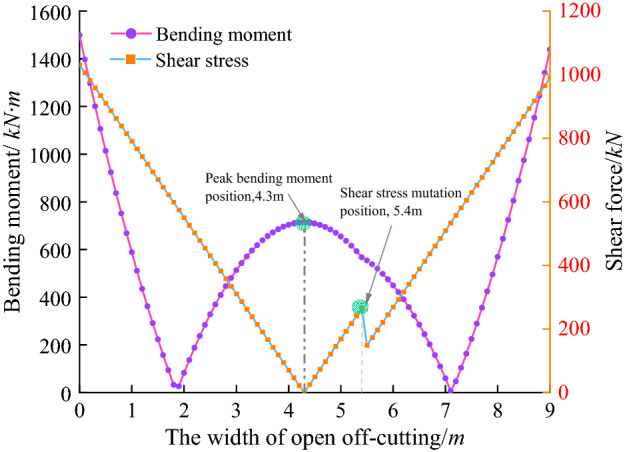
Distribution of bending moment and shear force along coal beam.

As shown in Fig. 5, the peak bending moment is located at both ends and the middle of the top coal. That is, the top coal both ends and the mid bottom are influenced by pull stress during the open-off cut excavation. Where through tension crack probably is generated, and rotation trend of the top coal towards the mined space is produced. After that, the stability of the damaged top coal is mainly controlled by the friction between the through cracks.

Influenced by the supporting effect of the single hydraulic prop in the open-off cut, the peak positions of bending moment and shear force are offset to the left side, which is a large distance side between the single hydraulic prop and the roadway side. The maximum peak values of bending moment and shear force appear at the position of *x* = 0. When the top coal does not produce tension cracks under the influence of the dead weight of the top coal, the following formula shall be met:

9 $$  \frac{{6M_{{\max }} }}{{bh^{2} }} = \left| {\frac{6}{{h^{2} }}\left[ {\frac{{F_{o} t^{2} }}{{lb}} - \frac{{F_{o} t^{3} }}{{l^{2} b}} - \frac{{Q_{k} l^{2} }}{{12b}}} \right] - \frac{{\gamma l^{2} }}{{2h}}} \right| \le \sigma _{t}   $$

The top coal does not slip along the through crack, the following formula needs to be met:

10 $$   \frac{{F_{{s\max }} }}{{bh}} = \left| {\frac{1}{h}\left( {\frac{{2F_{o} t^{3} }}{{l^{3} b}} - \frac{{3F_{o} t^{2} }}{{l^{2} b}} + \frac{{Q_{k} l}}{{2b}}} \right) + \frac{1}{{2b}}\gamma l} \right| \le \sigma \tan \varphi   $$

where: b is the width of the coal beam, taking the unit length is 1 m; $\sigma_{{\text{t}}}$ is the tensile strength of coal seam, taking 0.8 MPa, $\sigma$ is the horizontal stress, taking as 1.5 MPa.

**Figure 6:**
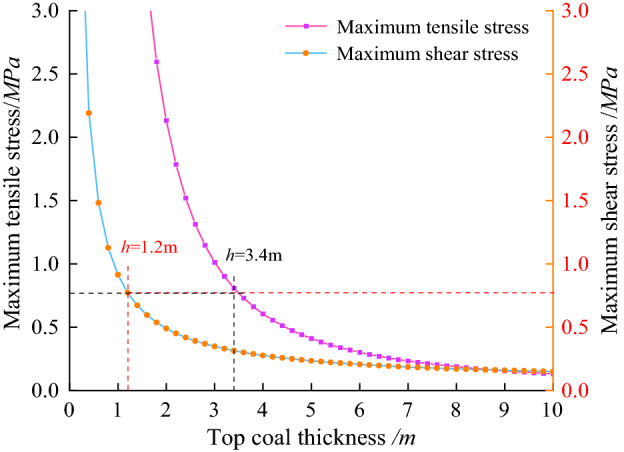
Variation of maximum tensile and shear stress with top coal thickness.

The relationship between the maximum tensile stress and shear stress and the thickness of top coal is shown in Fig. 6:

It can be seen from Fig. 6 that the maximum tensile stress and shear stress decrease rapidly with the increase of top coal thickness, indicating that the greater the top coal thickness, the more stable it is. However, the thickness of the top coal is too large, and the greater the undercover amount during the open-off cut excavation, which is unfavourable to the mining working face.

Therefore, it can be considered that the top coal does not produce through cracks, which can meet the production demand. Substituting the relevant parameters mentioned above into Eq. (9), (10). It is calculated that when the top coal thickness is greater than 3.4 m, the top coal does not produce through tension cracks. The broken top coal does not slide along the penetrating crack when the thickness of the top coal is greater than 1.2 m.

## Physical similarity simulation experiment of reasonable top coal thickness reservation

The lower 12,203 open-off cut is located under the gob, and the roof fissures are developed affected by the upper 12,203 working face mining. In addition, the section of the open-off cut is large, which is difficult to support, and roof caving is easy to occur. The top coal thickness of the lower 12,203 open-off cut is theoretically analysed in “Calculation of the reasonable top coal thickness” section to ensure the safety of cutting excavation and production. To determine the optimal top coal thickness, the deformation characteristics of different top coal thicknesses and the stability of surrounding rock of the large section cutting are to be investigated with a similarity simulation.

### Similarity model design

Based on the thickness of 1–2 coal seam and overlying strata are 9.96 m and 76 m. A similarity simulation experiment with length 3.0 m, width 0.2 m, and height 1.99 m is designed. The geometric similarity ratio, the bulk density, stress, and time similarity constant of the model are 1:50, 1.56, 78, and 7.1. Limited by the height of a simulation test frame, No.13 ~ 19 rock formations are not built above the 1–2 coal seam. The loading of lack rock formation is loaded with iron brick, so the actual height of the physics model is 1.26 m. Meanwhile, river sand, fly ash, mica, gypsum, white powder, and water are used to prepare similar materials in a certain proportion. The detailed ratio is shown in Table 1.

**Table 1:** Ratio of similar materials.

No	Lithology	Proportion number	Slice Thickness/cm	Total Thickness/cm	Gypsum/kg	Calcium carbonate/kg	Pulverized Coal/kg	Water/kg
12	Fine sandstone	837	3.1	126	0.9	2.2		2.77
11	Siltstone	728	12.2	122.9	2.8	10.8		11
10	Siltstone	728	1.5	110.7	0.3	1.4		1.39
9	Siltstone	728	6.9	109.2	1.5	6.3		6.21
8	Coarse Grained Sandstone	973	17.1	102.3	10.5	4.9		15.4
7	No.11 coal		5.1	85.2	1	5	19.8	4.6
6	Fine Grained Sandstone	837	25.8	80.1	8.1	18		23.4
5	Siltstone	728	7.2	54.3	1.5	6.3		6.3
4	No.12 coal		19.9	47.1	3.9	19.5	78	18
3	Siltstone	728	6.6	27.2	1.5	6		6
2	Fine Grained Sandstone	837	5.2	20.6	1.6	3.7		4.7
1	Siltstone	728	15.4	15.4	3.6	13.8		13.8

The thickness of the top coal is greater than 3.4 m, it is more stable based on the results in “Calculation of the reasonable top coal thickness” section. So, the top coal thicknesses of 3.0 m, 3.5 m, and 4 m are designed in the experiment, and the other parameters of the open-off cut are consistent with the in-situ. The die is embedded to the physical model forming the open-off cut during the model-built process. More detailed parameters about the open-off cut are shown in Table 2. The upper gob is formed by mining the upper 12,203 working face. Then, with the embedded mold extracted six times from the physical model, the excavated process of the open-off cut with different thickness of top coal is simulated.

**Table 2:** The designed parameters of the open-off cuts in the similarity model.

The open-off cut	Top coal thickness in-situ /m	Top coal thickness in the model /mm	Excavation depth of the floor in the model /mm
1	3.0	60	18.8
2	3.5	70	28.8
3	4.0	80	38.8

### Model monitoring program

**Figure 7:**
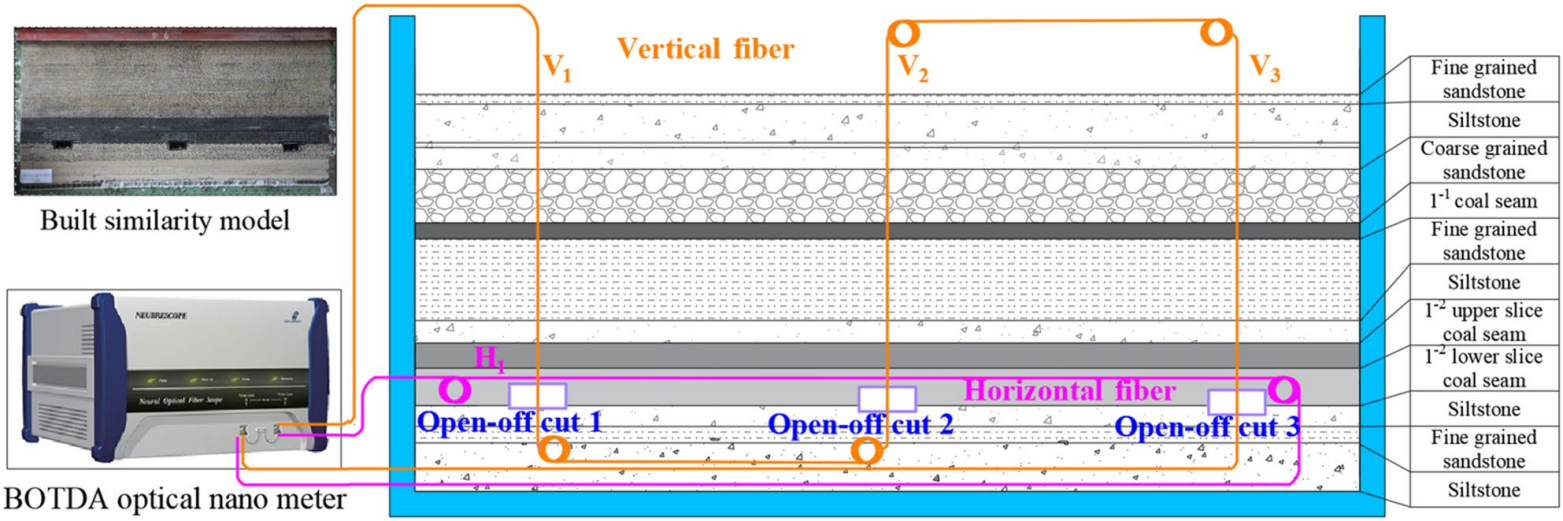
Schematic diagram of open-off cut layout.

To monitor the deformation characteristics of the surrounding rock of the open-off cut with different top coal thicknesses, the method of distributed optical fibre sensing (DOFS) based on BOTDA were utilized to monitor the internal deformation of the surrounding rock in the physical model experiment. An optical fibre with 2 mm diameter, named H1, was arranged horizontally in the top coal of the open-off cut in this experiment. Three vertical optical fibres same as horizontal fibre H1, named V1, V2, and V3, were arranged at the top coal centre of the open-off cut 1–3. The layout details of the test system can be found in Fig. 7.

An optical fibre signal demodulation equipment NBX-6055 was utilized in the experiment. The acquisition parameters of the instrument are set as follows: the distance range is 50 m, the sampling interval is 1 cm, the spatial resolution is 5 cm, and the average number is 2^16^. The test accuracy of the equipment is 7 με, and the data is collected after each excavation of the coal seam.

## Results

### The failure depth of top coal in the upper slice working face mining

**Figure 8:**
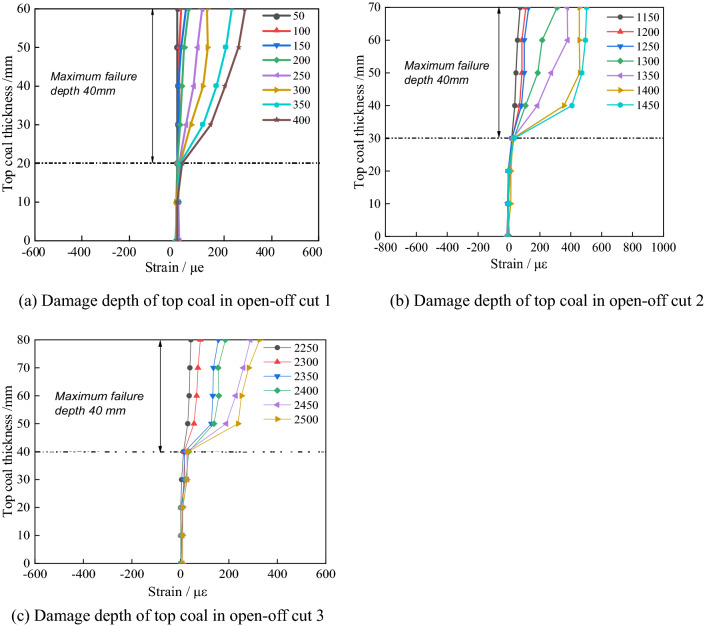
Damage depth of top coal in mining cut of upper layered working face.

The data of vertical fibre V_1_ when the working face is advanced from 50 to 400 mm, the data of vertical fibre V_2_ when the working face is advanced from 1150 to 1450 mm, and the data of vertical fibre V_3_ when the working face is advanced from 2250 to 2500 mm are extracted respectively, as shown in Fig. 8.

As is shown in Fig. 8, a gob is formed above the top coal of open-off cuts 1–3 with the mining of the upper layered working face. The stress is reduced with the top coal produced expanded deformation to the gob. The vertical optical fibre is pulled by in the expansion process of the top coal, resulting in positive strain. The damage extent and depth of top coal can be characterized by the magnitude and range of fibre strain. The damage depth of top coal gradually increases in the working face passing through the top coal position of open-off cuts 1–3. After the top coal completely enters the gob, the maximum damage depth is up to 40 mm.

### Vertical deformation characteristics of top coal

**Figure 9:**
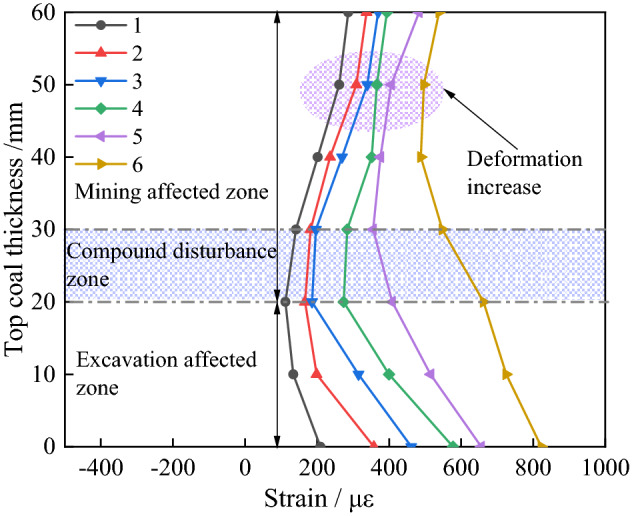
Deformation characteristics of top coal during excavation of the open-off cut 1.

The deformation characteristics of the top coal measured by the vertical optical fibre during the cutting excavation are shown in Fig. 9. During excavation, the vertical deformation of the open-off cuts 1 and 2 top coal can be divided into the mining influence area and the excavation affected area. There is a stable area with a thickness of about 0.5 m in the middle of the top coal in open-off cut 3. The top coal deformation area can be divided into the mining-affected area, stable area, and excavation-affected area.

The top coal thickness of open-off cut 1 is the smallest in the experiment. Affected by the excavation disturbance, the deformation of top coal gradually increases with the increase of the excavation times within the range of the top coal thickness. The deformation in the excavation-affected area is greater than that in the mining-affected area, and the coal below the mining-affected area is greatly affected by the excavation, and the subsidence increases obviously. As shown in Fig. 9, there is a composite disturbance affected area in the middle of the top coal. The maximum deformation in the mining-affected area reaches 548 με after excavation, and the excavation affected area reaches 822 με. The subsidence trend of top coal is obvious, and its stability is poor.

**Figure 10:**
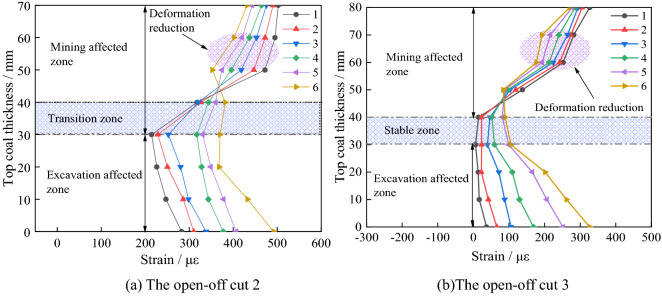
Deformation characteristics of top coal during excavation of open-off cut s 2 and 3.

As shown in Fig. 10a and b, during the excavation of open-off cuts 2 and 3, the coal body above the mining-affected area is progressively compacted by the gangue in the upper gob, and the deformation of the coal body shows a decreasing trend. It implies that the mining-affected area and the excavation-affected area of open-off cut s 2 and 3 are not interplayed, and there are some stable coal bodies in the middle of the top coal.

As shown in Fig. 10a, under the excavation effect, the deformation between 30 and 40 mm in the middle top coal of open-off cut 2 initially reduces and then rises. The zone is the transition area between mining and excavation influence. After the excavation is completed, the maximum deformation of top coal in the mining-affected area reaches 381 με, and the excavation affected area reaches 491 με. The subsidence trend of top coal is obvious, and it is comparatively stable.

During the excavation, the deformation is the same between 30 and 40 mm in the middle of the top coal of open-off cut 3. It indicates that there is a stable area with a thickness of about 10 mm in the middle of the top coal, which is relatively stable under the influence of mining and excavation. As is illustrated in Fig. 10b. The deformation in the mining-affected area of top coal is a relative decrease, under the influence the gangue gradually compacted in the gob. With the increase in excavation times, the deformation of top coal in the excavation affected area gradually increases. The maximum deformation of top coal in the mining-affected area reaches 90 με after the excavation is completed, and the excavation affected area reaches 326 με. The top coal deformation of open-off cut 3 in the excavation-affected area is only 39.6% and 66.3% of that in cut 1 and cut 2. The subsidence of top coal is small and the roof is more stable.

### Horizontal deformation characteristics of top coal

**Figure 11:**
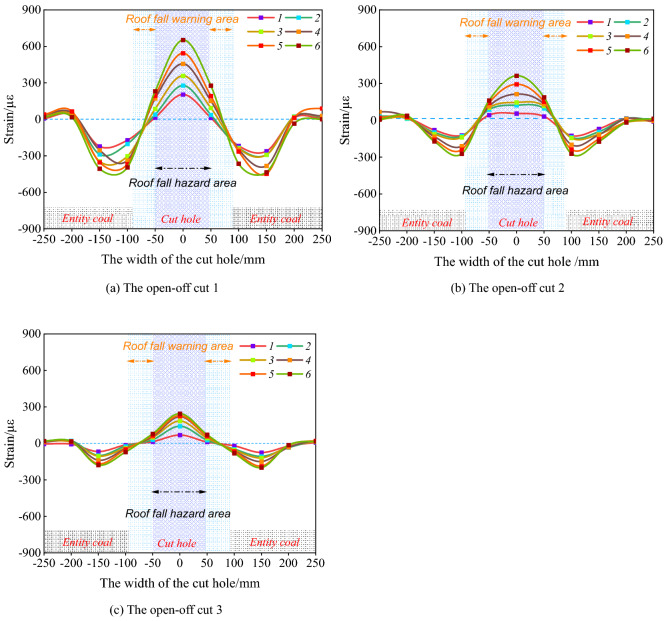
Deformation of open-off cut roof detected by distributed optical fiber.

During the excavation of the open-off cut 1–3, the horizontal deformation of the top coal with the optical fiber monitored was taken at the center of the width of the open-off cut top coal as 0 points, the positive and negative numbers represent the right and left positions of the open-off cut respectively. The abscissa in the figure is ± 250 mm, of which ± 100 mm is the open-off cut width, as shown in Fig. 11. The deformation of top coal presents a "W" type distribution.

According to the deformation characteristics of positive tension and negative pressure of optical fiber sensing, it shows that the middle part of the top coal has obvious subsidence under the influence of self-weight and the load of the overlying gob. With the continuous excavation of the open-off cut, the subsidence within ± 50 mm of the top coal center of the open-off cuts 1–3 are significantly greater than that on both sides, showing the zoning characteristics of large deformation in the middle and small deformation on both sides. It shows that when the open-off cut width is 9.96 m, the roof deformation is large within 5 m of the open-off cut center. If the support is not timely, the top coal has a large risk of falling. In this paper, the area within ± 50 mm of the top coal center is divided into a roof fall hazard area.

Within the range of ± 50-100 mm on both sides of the open-off cuts 1–3, the horizontal deformation results gradually change from positive strain to negative strain, indicating that the top coal subsidence gradually decreases near the two sides of the open-off cut . With the increase of the excavation distance of the open-off cut, the subsidence of the top coal around the open-off cuts 1–3 also gradually increases, but the peak deformation is only 7%-37% of the open-off cut center, and the stability is high. Therefore, within 2.5 m of the two sides of the open-off cut, the probability of top coal fall caving is small, and this area can be divided into a top coal caving early warning area.

### Deformation laws of top coal horizontal zoning

**Figure 12:**
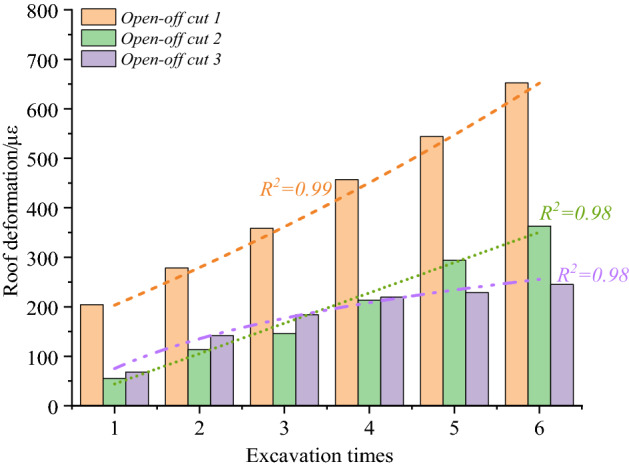
The deformation law of roof fall hazard area.

The deformation characteristics of the roof fall hazard area are shown in Fig. 12 during the excavation of open-off cuts 1–3. Compared to the optical fiber test results of the open-off cuts 1–3, the top coal center subsidence in the roof fall hazard area of the cut 1 is significantly greater than that of the open-off cuts 2 and 3 at each excavation stage. The roof fall hazard area deformation of the open-off cut 1 up to 652 με after the open-off cut is through. The deformation of the open-off cut 2 is 363 με. The deformation amount is about 56% of that of open-off cut 1. The deformation of open-off cut 3 is the smallest, and the subsidence of top coal after open-off cut through is only 228 με, it is about 35% of the open-off cut 1.

As shown in Fig. 12, it is found that the subsidence of the roof fall hazard area of the open-off cuts increases exponentially when the thickness of the top coal is 3 m. When the thickness of top coal is 3.5 m, the deformation in the roof fall hazard area in the cut is approximately linear. When the thickness of the top coal is 4 m, the deformation in the dangerous area of the top coal caving in the open-off cut increases logarithmically. It shows that when the open-off cut width is certain, the top coal thickness of 12,203 working face is set as 3 m, the stability of the top coal is poor, the subsidence of roof fall hazard area increases exponentially, the deformation of the top coal is large, and the maintenance of the cutting is difficult. The thickness of the top coal is set as 3.5 m, the stability of the top coal is moderate, the subsidence in the roof fall hazard area increases linearly, and the deformation of the top coal is in the middle. When the thickness of the top coal is set as 4 m, the stability of the top coal is higher, and the subsidence in the roof fall hazard area increases in an approximate logarithmic law. The deformation of the top coal changes little within a certain time after the open-off cut through.

**Figure 13:**
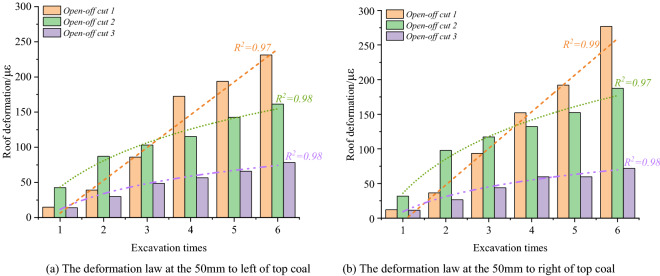
The deformation law of roof fall warning area.

The deformation law of the roof fall warning area of the open-off cut with different top coal thickness is shown in Fig. 13. The deformation characteristics of the roof fall warning area on the left side of the open-off cuts 1–3 are shown in the Fig. 13a, and the results of right side are shown in Fig. 13b. It can be found that with the increase of excavation times, the subsidence of the roof fall warning area on the left and right sides of the open-off cut 1 increases approximately linearly, and the deformation on the left side area of the open-off cut 1 reaches 231 με when the cut through, and the right area falling reaches 278 με. The subsidence of the roof fall warning area at the left and right sides of open-off cuts 2 and 3 rises logarithmically, and the growth rate of the top coal subsidence steadily declines after the third excavation. The subsidence of the area on the left side of open-off cut 2 after through is 161 με, 187 με on the right. The area on the left side of open-off cut 3 is 79 με, 72 με on the right. The roof fall warning area of open-off cut 2 and 3 is significantly smaller than that of open-off cut 1.

The above analysis shows that when the thickness of the top coal of the open-off cut is 3 m, the subsidence of the roof fall warning area increases linearly with the excavation of the open-off cut. The stability of the top coal is poor, and the probability of roof fall danger within a certain time after the cut is through is large. When the thickness is 3.5 m and 4 m, the subsidence of the top coal caving warning area increases logarithmically with the open-off cut excavation. The deformation in the top coal caving warning area obviously slows down during the fourth to sixth excavation, and the top coal stability is good.

By comparing Fig. 13a,b, under different top coal thicknesses, along the open-off cut width direction, the deformation of roof fall hazard area during the open-off cut excavation is significantly greater than that of roof fall warning area. The thickness of the top coal is 3 m, the deformation law of the top coal changes from exponential to linear deformation from roof fall hazard area in the center to roof fall warning area on both sides. The stability of the top coal is poor and the risk of roof fall is high. When the thickness of the top coal is 3.5 m, the deformation law of the top coal changes from linear to logarithmic deformation from roof fall hazard area in the center of the top coal to the roof fall warning area on both sides. The top coal shows a certain self-stability, and the stability is good. When the thickness of the top coal is 4 m, within the width of the open-off cut , whether it is in the roof fall hazard area or the warning area, the subsidence law of the top coal is a logarithmic deformation law. The top coal shows a good self-stability ability, and the stability is higher.

## Discussions

### Determination of reasonable top coal thickness

According to the experimental results, the thickness of the top coal in the large section open-off cut under the gob is increased from 3.0 m to 4.0 m, and the subsidence deformation law in roof fall hazard area changes from the exponential to the linear, and finally turns to the logarithmic subsidence law. It will take some time for shunting between the continuous excavator and the anchor bolt machine after the excavation of the open-off cut in-situ. There is an empty roof period in a certain range in front of the open-off cut. When the thickness of the top coal is 3 m, the subsidence amount and speed of the top coal are large, which is not conducive to on-site safety. When the thickness of the top coal is 4 m, the roof fall hazard area sinks in a logarithmic relationship. The subsidence of the top coal is small, which has a good self stabilization ability. The strong support method is adopted on site, and the stability of the open-off cut is the highest. However, there are shortcomings of large undercover depth and waste of coal resources. When the thickness of top coal is 3.5 m, the roof fall hazard area sinks with linear deformation law, and the stability of top coal can be guaranteed after strong support. It can not only reduce the undercover amount, but also improve the coal recovery rate. To sum up, when the thickness of top coal is 3.5 m, it is not only conducive to the stability of top coal, but also conducive to on-site construction and improves the resource recovery rate.

### Field observation on stability of top coal in the 12,203 open-off cut

**Figure 14:**
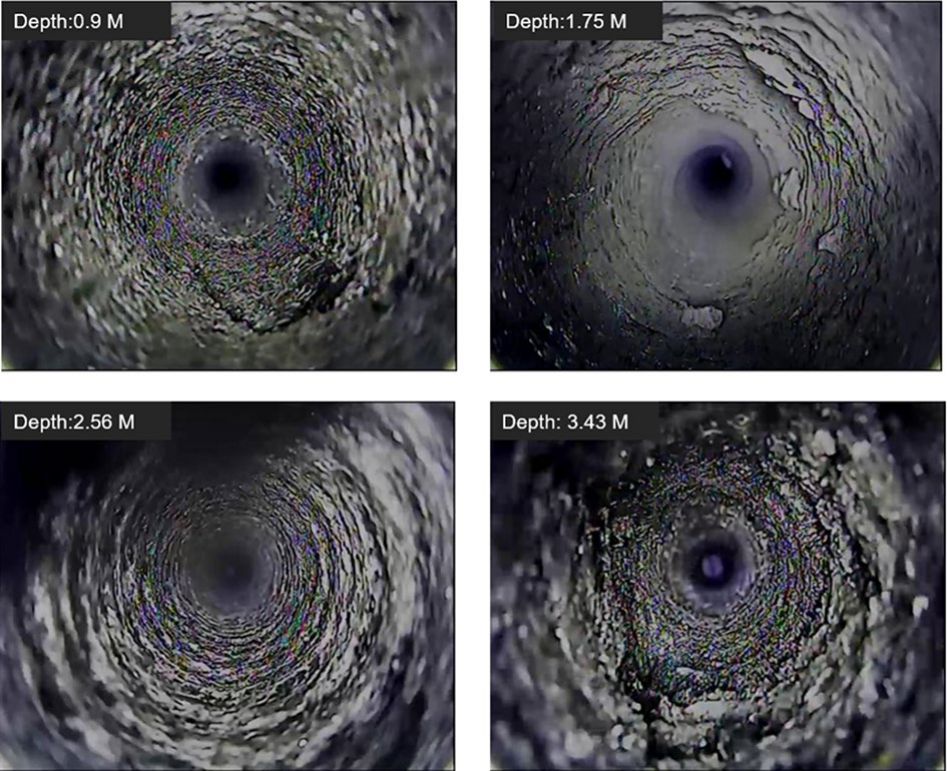
Top coal integrity on 12,203 open-off cut.

**Figure 15:**
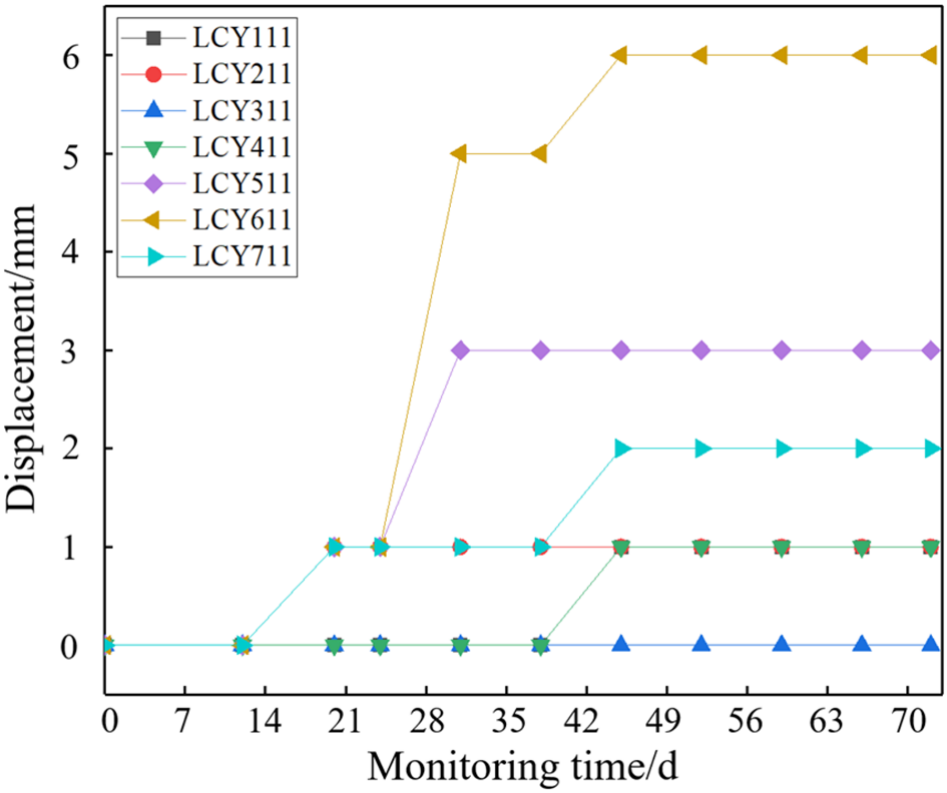
Observation results of roof layer separation instrument.

The reserved thickness of top coal is 3.5 m on site. The deformation of the top coal in the 12,203 open-off cut is observed through the borehole peep and the roof separation instrument. As shown in Fig. 14, there are some small horizontal fracture near 0.9 m of the top coal. There are some coal chips at 1.75 m and 2.56 m. With small fracture development, the top coal at 3.43 m is relatively broken due to the influence of the overlying gob. The monitoring results of the roof separation instrument are shown in Fig. 15. The maximum separation inside the top coal is 6 mm after 70 days of monitoring. The results of comprehensive borehole peeping and roof separation monitoring show that the top coal is relatively stable when the top coal thickness is 3.5 m under the condition of support.

## Conclusions

1. Based on the theoretical calculation results, the thickness of top coal in large section open-off cut of 12,203 working face in Huojitu minefield is greater than 3.4 m, there is no tension crack, and the thickness is greater than 1.2 m, the top coal does not slip along the through crack.
2. The top coal deformation of the open-off cut is affected by both mining and excavation disturbance in the vertical direction, which can be divided into mining disturbance area and excavation disturbance area. The thickness of top coal is 3 m, there is a complex disturbance zone in the middle of the top coal, and the stability is poor. The thickness is 3.5 m, there is a mining and excavation disturbance transition zone in the middle of the top coal. The thickness is 4 m, there is a certain thickness of stable zone in the middle of the top coal, and the stability is good.
3. The distributed optical fiber monitoring system has high sensitivity and accuracy in the model experiment and can monitor the internal deformation of the model well. According to the horizontal optical fiber monitoring results, it is innovative found that the deformation of the top coal in the width direction of the open-off cut are to be divided into roof fall hazard area and warning area. In the process of cut hole excavation, the deformation law of top coal in the roof fall hazard area changes from logarithmic to linear deformation, and finally to exponential deformation law. The deformation law of top coal in the roof falling warning area changes from linear deformation to logarithmic deformation.
4. The thickness of the top coal of the cut is 3.5 m, the top coal in the roof fall hazard area increases linearly with the increase of excavation times, and the stability is moderate. It is comprehensively determined that 3.5 m top coal is reserved on site. After support, the top coal has good integrity, and the maximum layer separation is 6 mm, which can meet the production requirements.

This paper focuses on the stability of the top coal in the case of large section open-off cut with different top coal thickness without support, and does not consider the influence of support on the top coal deformation. It is the focus of future research to carry out the reasonable thickness of large section open-off cut top coal with different support methods and analyze its deformation law.

## Data Availability

The data used to support the findings of this research are included within the paper.
